# Gene Therapy: A Paradigm Shift in Dentistry

**DOI:** 10.3390/genes7110098

**Published:** 2016-11-10

**Authors:** Nida Siddique, Hira Raza, Sehrish Ahmed, Zohaib Khurshid, Muhammad Sohail Zafar

**Affiliations:** 1 Department of Oral Biology, Bahria University Medical and Dental College, Karachi 75260, Pakistan; nidasiddique@hotmail.com (N.S.); raza.hira@gmail.com (H.R.); dr.sehrishahmed1@gmail.com (S.A.); 2 Department of Biomedical Engineering, College of Engineering, King Faisal University, Al-Hofuf 31982, Saudia Arabia; drzohaibkhurshid@gmail.com; 3 Department of Restorative Dentistry, College of Dentistry, Taibah University, Madina Munawwarrah 41311, Saudi Arabia

**Keywords:** gene therapy, pain, cancer, DNA vaccination, bone repair, tooth repair, orthodontic tooth movement

## Abstract

Gene therapy holds a promising future for bridging the gap between the disciplines of medicine and clinical dentistry. The dynamic treatment approaches of gene therapy have been advancing by leaps and bounds. They are transforming the conventional approaches into more precise and preventive ones that may limit the need of using drugs and surgery. The oral cavity is one of the most accessible areas for the clinical applications of gene therapy for various oral tissues. The idea of genetic engineering has become more exciting due to its advantages over other treatment modalities. For instance, the body is neither subjected to an invasive surgery nor deep wounds, nor is it susceptible to systemic effects of drugs. The aim of this article is to review the gene therapy applications in the field of dentistry. In addition, therapeutic benefits in terms of treatment of diseases, minimal invasion and maximum outcomes have been discussed.

## 1. Introduction

Gene therapy deals with replacing the defective genes with their correct analogues to produce functional proteins. Evidence suggests that gene therapy can be used to prevent, alleviate or cure underlying disorders including cancers, infectious diseases, and genetic and autoimmune disorders [1]. A gene therapy medicinal product is defined as any biological product consisting of an active component of recombinant nucleic acid used to regulate, repair, replace, enhance or delete a genetic component. However, vaccines against infectious diseases are not classified as gene therapy medicinal products [2]. In terms of functioning, every gene stores information for synthesis of a specific protein that in turn controls various bodily functions and mechanisms. Any defect in the gene, therefore, results in an inevitable aberration in the physiology and results in development of diseases [1]. Gene therapy is being considered as an innovative treatment modality for the correction of faulty genes by genetic modification of the human cells and subsequent formation of functional proteins.

Gene therapy is a two-step process; in the first step, human genetic coding for the therapeutic protein is first cleaved and inserted into the genome of a carrier or vector that is usually an attenuated virus. In the second step, the entry of modified vector to the target human cells results in releasing the DNA sequence that becomes integrated within the chromosome. As the gene is “switched on” in its correct location, the cells with the new genetic design start forming the required therapeutic proteins [1,3,4]. In general, the gene therapy can be classified into two distinct stages [2]—somatic and germ line gene therapy.

The somatic gene therapy involves changes in the target cells, however, that are not transferred to the next generation. In contrast, with germ line gene therapy, the modified genes are transferred to the next generation. There are certain ethical issues regarding germ line gene therapy; hence, somatic cell gene therapy is currently allowed [2]. Although germ line gene therapy offers possible treatment for hereditary disorders in the future, due to ethical issues, it has been limited to animal models. Besides ethical concerns, the risk of unexpected damage to the developing fetus [5] is also a major concern obstructing the use of germ line gene therapy for human clinical trials.

**Figure 1:**
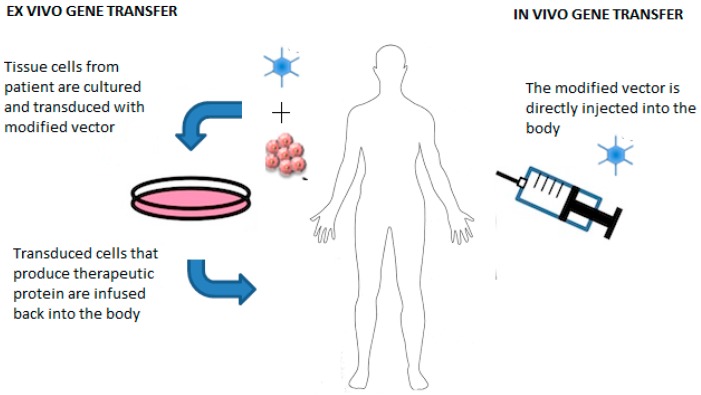
Schematic presentation of various approaches used for the delivery of vectors and genes for genetic therapy.

Depending on the delivery method of the vector, gene transfer can be achieved via two techniques (Figure 1)—in vivo gene transfer that engages direct injection of genetically engineered vectors into the patient, or ex vivo gene transfer that involves injection of the genetically engineered vector into cultured tissue cells followed by transplantation of the altered tissues into the body [4,6]. The characteristics of an ideal vector include high specificity, low virulence and its ability to completely unload the normal human gene into the host cells [7]. Viral vectors are more efficient in transferring the gene but carry a risk of causing illness. Most popular viral vectors include adenoviruses and retroviruses [3,8]. Non-viral vectors are cheaper, safer and can deliver large amounts of DNA into the host cell. However, efforts are being made to improve their transfection rate.

Plenty of research and clinical trials in the last couple of decades have resulted in a better understanding of gene transfer vectors and improved clinical outcomes [2]. Currently, gene therapy has emerged as a major medical breakthrough and a number of researchers have focused on using modern advancements in genetic therapy to treat life-threatening and refractory diseases such as prevention of HIV [9], management of hematological disorders [10], metabolic disorders [11,12], cancer treatment [13,14,15] and stem cell approaches [16]. Since the introduction of gene therapy for dental applications in the 1990s [4], progressive clinical trials are being carried out in humans and on animal models for various applications with promising outcomes. The aim of this paper is to review the applications of gene therapy for various oral and dental conditions. In addition, related current and future prospects have been discussed.

## 2. Historical Background

The concept of genetic therapy has been known for many decades. The key developments in the field of genetic therapy have been shown in Table 1. The idea of transforming principles was introduced by Griffith in 1928 during his experimentation on pneumococcal bacteria. The role of deoxyribonucleic acid (DNA) to purify the transformed genetic products was described in 1944 followed by the idea of transduction through transformation of genetics in bacteria in 1952. During the 1950s and 1960s, researchers focused on understanding the double helix structure and role of DNA for genetic therapy. Further research led to conduction of the first ever human gene therapy trial in 1973. In 1990, the Food and Drug Administration (FDA) approved gene therapy for therapeutic applications in the United States. In the last decade (2001–2010), a number of genetic products such as Gendicine™ (SiBiono GeneTech, Shenzhen, China ) and Cerepro^TM^ (Ark Therapeutics Ltd, London, UK) have been developed and approved for therapeutic applications and have become commercially available (Table 1). Further research is in progress to develop more products for therapeutic applications focusing particularly on malignant lesions and autoimmune diseases.

## 3. Applications in Dentistry

**Figure 2:**
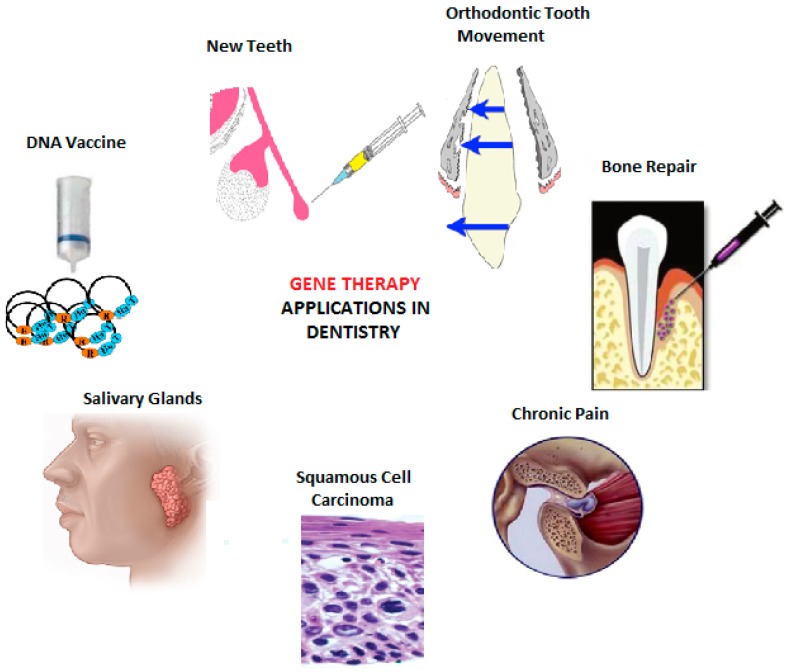
Current and potential applications of gene therapy in the field of dentistry.

Since the introduction of gene therapy for dental applications [29], remarkable progress has been made in the field of genetic therapy for a range of applications in dentistry (Figure 2). In order to improve the quality of life, gene therapy has promising outcomes for potential treatment for multiple disorders and has been discussed in this review.

### 3.1. Orofacial Pain

Orofacial pain refers to the pain associated with hard and soft tissues of the face, head and neck region. The pain impulses are conducted through the 5th cranial (trigeminal) nerve to the central nervous system. Due to the diffuse and referral nature, the diagnosis and management of orofacial pain remains a major challenge. Patients commonly seek treatment for common causes of orofacial pain such as temporomandibular disorders (TMD) [30].

**Figure 3:**
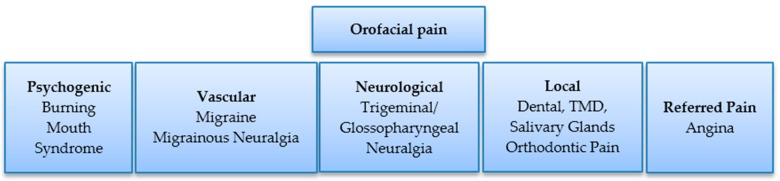
Common causes of pain in the oral, dental and facial tissues [40].

The orofacial pain may originate from dental hard tissues (pulpitis, hypersensitivity), oral soft tissues (Temporomandibular joint, glands, orthodontic), neurological tissues (neuralgia) and vascular or psychogenic tissues (Figure 3). Conventionally, the pain management involves using analgesics [30,31,32] and sedatives [33]. Gene therapy is being investigated for improving management of chronic pain by sparing the use of drugs with the associated risk of systemic toxicity, opioid addiction and other side effects [34]. The continuous production and secretion of anti-nociceptive proteins in or near spinal dorsal horns may be achieved in two ways. Firstly, the modified adenovirus, adeno associated virus (AAV) or lipid encapsulated plasmids coding for Interleukin-10, a therapeutic protein, may be injected into the sub arachnoid space to transduce the pia mater cells [35]. Secondly, a modified herpes virus may be introduced into the nerves of Dorsal Root Ganglia (DRG) via an intradermal injection to the skin. The rationale for using the herpes virus is that it infects nerves, and it therefore has the ability to travel to the DRG via nerve endings in the skin. In the DRG, it codes for an inhibitory neurotransmitter, an anti-inflammatory peptide or decreases the synthesis of an endogenous nociceptive molecule that results in alleviation of pain [35,36]. At present, the use of gene therapy for alleviation of pain is mainly limited to animal models. Recently, a reduction in trigeminal pain has been reported encoding the human preproenkephalin gene through a herpes simplex vector in a mouse model [37]. Gene therapy may offer a dose of hope to the pain therapist in treatment of pain syndromes such as trigeminal neuralgia and temporomandibular joint disorders with improved vector systems in the future [38,39].

### 3.2. Carcinomas

Squamous cell carcinoma of the head and neck (SCCHN) region includes cancer of the oral cavity, paranasal sinuses, larynx, pharynx and skin of the head and neck. It is regarded as the 6th most common cancer in the world [41]. Genetic disorders are commonly associated with odontogenic tumors [42]. The conventional treatment approaches and ongoing advances in surgical, radiotherapy and chemotherapeutic measures for SCCHN did not show any remarkable improvement in the five-year survival rate [7].

**Figure 4:**
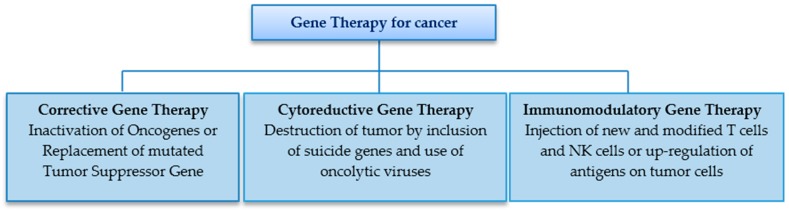
Various strategies and types of gene therapy approaches used for cancer treatment.

Gene therapy holds the potential to give exciting treatment results and improved prognosis. Figure 4 shows various strategies and types of gene therapy approaches used for cancer treatment. It has been seen that the response rate is higher in patients receiving a combination of gene therapy with other conventional treatment modalities. Carcinogenesis occurs by either an overexpression of oncogenes such as Ras, Myc, Bcl-2, ErbB2 or an underexpression or mutation of tumor suppressor genes such as p53, p16, p21, Rb genes [41,43]. It is also a convenient procedure to carry out due to the ease of access. Injection into the localized tumor mass precludes unwanted side effects on the body and premature degradation of the gene before it reaches the target cells [3,44]. Currently, there are more than 1000 clinical trials of gene therapy in progress worldwide, out of which more than 700 are related to cancers including 54 for SCCHN [45]. The most common viral vectors being used in this regard are adenoviruses and retroviruses [46].

In terms of cancer gene therapy, it is vital to consider the following points:

- Corrective gene therapy (Gene Replacement Therapy) involves correction of the underlying genetic defect to control the unrestricted multiplication of tumor cells. The over-expressed oncogenes are blocked or silenced by inclusion of DNA into the cell, while successfully disrupting the processes of transcription and translation. It is also worth mentioning that the mutation or inactivation of the p53 gene is seen in the infancy of many tumors, due to which the defensive mechanism of apoptosis is disabled and the abnormal cells are free to multiply. With gene therapy, the correct copy of p53 gene is introduced into the tumor cells leading to apoptosis. At the same time, this makes the tumor cells more receptive to radiotherapy [41].
- In 2003, the first gene therapy drug Gendicine, a recombinant human adenovirus p53, was formulated and approved by the Chinese authorities for the treatment of SCCHN and other cancerous lesions. In a randomized clinical trial, 135 SCHNN patients were treated with radiotherapy alone or in combination with Gendicine. The majority of patients (64%) receiving both Gendicine and radiotherapy exhibited complete remission unlike the radiotherapy only group that showed remission ~19% [26,47,48].
- Cytoreductive gene therapy aims at destruction of tumor cells in multiple ways—for example, through the insertion of suicide genes into tumor cells which encode for enzymes that convert the chemotherapeutic drugs into their toxic form [7]. The introduction of genes can also limit angiogenesis and increase apoptosis in tumor cells .The highlight of this approach, however, are the oncolytic viruses which selectively multiply in tumor cells and kill them. These can significantly reduce the size of the tumor after its surgical removal and prevent metastasis [7]. In 2005, the first genetically engineered oncolytic virus, H101 Adenovirus, was approved for treatment of SCCHN in China [49]. Advanced stage cancer patients showed a 79% response rate with both chemotherapy and the modified adenovirus, as compared to a 40% response rate with chemotherapy alone [50]. These results suggested the promising outcome of using gene therapy as an adjunct for the management of cancers.
- Another approach aims at the modification of the immune system. This is designed to boost up a host′s immune system by injecting genetically modified hematopoietic stem cells and T cells that are highly efficient in identifying and killing tumor cells. This may be coupled by insertion of a gene in tumor cells to upregulate their antigen markers and make them more susceptible to destruction by the body′s own immune system. The concentration of cytokines in tumor cells may also be increased by insertion of a gene encoding for cytokines. Immunotherapy may be beneficial in treatment of SCCHN, melanoma, lymphoma and some virus induced malignancies [51].

Currently, gene therapy for SCCHN is evolving through clinical trials. Its progression to clinical applications in combination with conventional modalities will make a mark in improving the survival rate of patients, especially those with refractory or recurrent diseases. The treatment of metastatic diseases is, however, challenging due to the risks involved with systemic administration of gene therapy agents.

### 3.3. Bone Repair

Unlike other dental hard tissues (such as enamel dentin), bones can be remodeled and have a good potential to regenerate and repair [52,53]. In many instances, bone injuries and fractures heal without scar formation. Nevertheless, in the case of pathological fractures or massive bone defects, bone healing and repair may be challenging [41,54,55,56]. At least four imperative elements are required for successful bone regeneration, namely osteoinduction, differentiation of osteoblasts leading to production of the osteoid matrix, osteoconduction and mechanical stimulation. Gene therapy enhances the first three conditions [4].

Bone morphogenetic proteins (BMP-2, 4 and 7) are the only signaling molecules that can singly induce de novo bone formation at orthotopic and heterotopic sites. The osteoinductive potential of BMPs makes them clinically valuable as alternatives to bone grafts [56]. In order to achieve healing of mandibular osseous defects, an in vivo study demonstrated the possibility of delivering the BMP-2 genes directly to the tissues via an adenoviral vector [57].

Moreover, in another piece of in vivo research, several different cell types—such as non-osteogenic fibroblasts (from human gingiva and dental pulp) and myoblasts, as well as osteoblasts—can express the BMP-7 gene after being infected with an adenoviral vector. These cells then are able to differentiate into bone forming cells when placed in an osseous defect in vivo [58]. Although BMPs can cause osteoinduction individually, there is strong evidence that these factors work in collaboration to induce bone formation. For example, coordinated expression of BMPs 2,3a, 4, 7 and 8 during fracture healing is important in both skeletal development and repair [59].

Platelet derived growth factor (PDGF) is another potent mitogen that plays a pivotal role in wound healing. PDGF exerts its biological effects on cell migration, proliferation, and synthesis of extra cellular matrix, and is anti-apoptotic in nature. Its activity is arrested by the growth arrest gene (gas gene). The advent of the bioactive PDGF gene has helped us to overcome the inhibitory effects of the growth arrest gene, which is critical for wound healing [60]. The bone sialoprotein is a significant non-collagenous protein involved in bone repair along with the CBFA1 gene, which is involved in cell differentiation and gene expression of bone sialoprotein during bone repair and regeneration [61].

### 3.4. Salivary Glands

Salivary glands secrete saliva that has a physiological role for lubrication, mastication and digestion of food [62]. Saliva is rich in antimicrobial peptides that are a vital component of local immunity [63,64,65]. The lack of salivary gland functions results in dryness of the mouth (xerostomia). Common etiological factors of xerostomia included salivary gland impairment, radiotherapy of the head and neck, autoimmune disorders (such as Sjogren′s syndrome) and certain medications [66]. Salivary glands exhibit several important features such as: self-containment due to a surrounding capsule that is likely to minimize the undesirable access of administered vectors and transgenes to other tissues [1], and highly efficient protein production [58]. Their ability to secrete proteins into the bloodstream makes them potentially useful target sites for gene transfer in a minimally invasive manner with the help of intraductal cannulation.

With the origin of Aquaporin 1 gene, it is anticipated that patients suffering from hypofunctional salivation due to ionized radiation will soon be cured with the help of gene therapy [67]. These ionized radiations cause severe damage to the fluid secretory portion (acinar cells) of the salivary gland that lies in the field of radiation [68]. Aquaporin 1 (AQ1) is a water channel protein that counterbalances this detriment by a constitutively activated water channel. In the irradiated submandibular glands of a rat and the in parotid glands of an adult rhesus monkey, it showed positive results [58]. Recently, Lai et al. reported positive results while using aquaporin gene therapy to treat the Sjögren′s syndrome symptoms in animal modelling [69]. Although the concept of gene therapy for salivary glands was introduced more than 20 years ago [70], the first ever clinical human trial was reported in 2012 [71]. Baum et al. [71] reported promising results for using aquaporin-1 cDNA for the management of radiation-related salivary hypofunctions during clinical trials. This has been considered as a translational step, as this approach can be used to treat a huge number of patients suffering from dry mouth (xerostomia) due to radiation therapy and autoimmune diseases. A recent clinical trial has advocated the safety of using AQP1 gene therapy for the management of xerostomia patients [72]. Patients undergoing radiotherapy usually develop xerostomia due to irreversible acinar damages. The successful human clinical trials illuminated hopes for clinicians to overcome radiotherapy related salivary hypofunctioning in the near future. However, the use of AQP1 gene therapy for the management of radiotherapy related xerostomia may involve a few challenges such as prevention of host immune reaction and repeated administrations throughout the course of treatment [72]. Further clinical trials are required to be conducted to decide if it is useful to pursue the AQP1 gene transfer strategy clinically.

Gene therapy has been augmented in the treatment of salivary glands along with other systemic conditions. Secretory gene proteins are injected into salivary glands, which are secreted in an exocrine manner. This gene transfer mechanism results in treating disorders of the mouth and upper gastrointestinal tract. Researchers have injected a naturally occurring salivary anti-candidal polypeptide human histatin 3 (H3)) into the azole resistant candidiatic submandibular gland of rats. Copious amounts of H3 polypeptide were secreted in saliva that killed both azole-sensitive and azole-resistant Candida species with approximately equal efficiency [73].

Gene transfer presents an attractive opportunity to correct the systemic single-protein disorders, for example growth hormone deficiency in children. For many years, researchers have tried to accomplish the secretion of a transgene product into the blood stream from the exocrine salivary glands [74]. This has finally been achieved by the transfer of the human growth hormone gene (hGH) via a recombinant adenovirus administered into rat salivary glands [3,66]. The subsequent results showed an increase in serum hGH levels, indicating the endocrine function of the exocrine salivary glands [1]. The identification of genes involved in the salivary gland lesions is the main challenge in the progress of gene therapy for these lesions.

### 3.5. Orthodontic Tooth Movements

Orthodontic tooth movement is possible due to remodeling of periodontal ligament and alveolar bone that is controlled by osteoclasts and osteoblasts. Precursors of osteoclasts are hemopoietic cells, whereas osteoblasts originate from stromal cells. Maturation and activation of osteoclasts require interaction with cells from the osteoblastic lineage. The molecules mediating such interactions are the receptor activator of the nuclear factor kappa B (RANK) or receptor activator of nuclear factor kappa-B ligand (RANKL) Osteoclastic precursors express on their surface RANK, the receptor for RANKL that binds and converts them into multinucleated giant cells. Osteoprotegerin (OPG), a soluble receptor produced by osteoblasts, is in competition with the RANK receptor binding to RANKL. Upon binding with RANKL, it inhibits osteoclastogenesis, thus jamming the process of bone resorption. Two significant studies were made by using gene therapy with OPG and RANKL to speed up and impede orthodontic tooth movement in a rat model. Local RANKL gene was transferred to the periodontal tissue, which resulted in accelerated orthodontic tooth movement by approximately 150% after 21 days, without evoking any systemic effects, thereby reducing the time of treatment. It was suggested that Local RANKL gene transfer might be a useful tool not only for shortening orthodontic treatment, but also for moving ankylosed teeth. In contrast to RANKL, local OPG gene transfer inhibited tooth movement by about 50% after 21 days of application of force. This will cause a paradigm shift in orthodontic treatment by reducing treatment time with improved results [58]. In addition, gene therapy has shown promising results for controlling the pain of orthodontic tooth movement [32]. Further research may result in the development of gene therapy therapeutic products that can be prescribed in the future to control the pain of orthodontic tooth movement.

### 3.6. Tooth Repair and Regeneration

Tissue engineering has been developed for the regeneration and repair of tissues Research has reported promising outcomes for the tissue engineering of various oral and dental tissues [53,75]. Gene therapy presents an attractive concept of restoring the oral tissues lost due to caries, periodontal diseases and trauma. This could widen the scope for development of new teeth—the biological implants for missing teeth. This makes use of the two basic approaches including in vivo and ex vivo gene therapy. In vivo gene therapy, the healing potential of tissues such as dentine pulp complex, is enhanced by genes stimulating dentine formation after being applied directly on the exposed dental pulp [76]. Many people have supernumerary teeth that arise from the third set of dentition. This third dentition can also be induced to form teeth in a natural way by turning on or activating genes which code for proteins and signaling molecules making up the basic structure of teeth [77].

The ex vivo gene therapy is based on multipotent dental stem cells that have the potential to differentiate into any tissues including dental tissues. Their sources include dental pulp, apical papilla, dental follicles, deciduous teeth, and periodontal ligaments. Stem cell based genetically engineered cells are cultured, modified or transfected, and then re-implanted back into the recipient. Successful regeneration of the periodontal attachment apparatus including alveolar bone and cementum has been accomplished using a combination of stem cells engineered by the adenovirus to express the BMP-2 gene [78]. Transfected pulp stem/progenitor cells can also differentiate into odontoblasts, which are then transplanted on the exposed pulp [76]. Similarly, a tooth germ can be created in vitro or ex vivo with a culture of epithelial and mesenchymal stem cells. The tooth germ is implanted into the alveolar bone to develop into a fully functional, non-metal tooth implant [79]. Scientists are discovering the 20 basic proteins that are essential in tooth development. The gene therapy has shown to control the differentiation of stem cells [80]. For instance, the repetitive administration of gene therapy decreases as stem cells have the ability to self-renew. Recently, promising results have been reported for cartilage tissue engineering using a gene expression system [81]. A combination of various vectors of osteogenic genes has been added to bone tissue engineering scaffolds. In addition, osteogenic genes are reported to promote the cellular differentiation and bone formation using tissue engineering approaches [82].

## 4. Conclusions

Gene therapy has emerged as an active area of research for a range of biomedical and dental applications. Considering the exponential rise in reported cases of oral squamous cell carcinoma and periodontal diseases, gene therapy is expected to be a very useful tool for the management of oral diseases and improving the prognosis and quality of life. The successful outcomes of human clinical trials in recent years have ignited hopes for clinicians for the progression of gene therapy to practical applications very soon. Although previous research has reported very favorable results, additional in vivo studies and clinical trials are essential before translating the gene therapy to clinical applications for oral and dental conditions.

Irrespective of plenty of active research and ongoing clinical trials, there are a number of limiting factors such as technique sensitivity, identification of related genes and vectors, delivery of genes at the site of action and duration of action. Additionally, the transfer of a large amount of genes into many cells is crucial to achieve the desired therapeutic effect and may not be cost-effective. In addition, there are ethical and safety issues for using gene therapies in humans; only somatic cell gene therapy is allowed at present. Many disorders, especially cancers, emanate from mutations of more than one gene. Researchers have yet to unravel the genetic basis of such diseases in order to tailor a more precise cellular therapy. Improved and efficient vector systems with reduced toxicity and a higher transfection rate are instrumental in successful treatment and good prognosis. Research should be channelized into discovering ways to overcome these challenges that limit gene therapy from becoming a mainstream treatment modality. This can be expected to overcome the obstacles associated with the clinical applications of gene therapy in the near future. Further research and developments will direct a new dimension with dentists emerging as “gene therapists” and excelling in repairing alveolar bone defects and treating oral cancer in the clinical setups.

## Figures and Tables

**Table 1:** Chronological advancements in gene therapy approaches.

Year	Key Advancements	Ref.
1928	Griffith′s experiments with pneumococcal bacteria and introducing transforming principles	[17]
1944	Purification of transforming substance; first reported deoxyribonucleic acid (DNA) causes the transformation	[18]
1952	Transduction (transfer of genetic through bacteria) was introduced for the first time	[19]
1953	Double helix structure of DNA was described	[20]
1961	It was reported that viral infections can inherit genetic mutations	[21]
1962	First ever DNA-mediated heritable transformation of a biochemical trait	[22]
1973	First gene therapy trial conducted in humans	[23]
1989	Reported gene transfer in humans	[24]
1990	FDA approved gene therapy trial in humans for therapeutic applications.	[2]
1995	Gene therapy introduced for dental applications	[4]
1999	Jesse Gelsinger died during a clinical trial of gene therapy	[25]
2003	China approved gene therapy for clinical applications	[2]
2005	Gendicine™ (an adenoviral vector) approved for the treatment of squamous cell carcinoma	[26,27]
2009	Cerepro® (an adenoviral vector) gene therapy for the treatment of malignant brain tumors	[28]
2012	A gene therapy product (Glybera) that is an adeno-associated viral vector was recommended for the European Union	[2]
